# Rectal deformation management with IGRT in prostate radiotherapy: Can it be managed with rigid alignment alone?

**DOI:** 10.1002/acm2.14241

**Published:** 2024-01-09

**Authors:** Mitchell Polizzi, Elisabeth Weiss, Nuzhat Jan, Anthony Ricco, Siyong Kim, Alfredo Urdaneta, Mihaela Rosu‐Bubulac

**Affiliations:** ^1^ Department of Radiation Oncology Virginia Commonwealth University Richmond Virginia USA

**Keywords:** IGRT, online registration, prostate alignment, rectal deformation, rigid registration

## Abstract

**Purpose:**

It is challenging to achieve appropriate target coverage of the prostate with Image Guided Radiation Therapy (IGRT) while simultaneously constraining rectal doses within planned values when there is significant variability in rectal filling and shape. We investigated if rectum planning goals can be fulfilled using rigid CBCT‐based on‐board alignment to account for interfraction rectal deformations.

**Methods:**

Delivered rectal doses corresponding to prostate alignment (“PR”) and anterior rectum alignment (“AR”) for 239 daily treatments from 13 patients are reported. Rectal doses were estimated by rigidly mapping the planned dose on the daily CT derived from the daily CBCT according to respective alignment shifts. Rectum V95% (rV95%) was used for analyses.

**Results:**

Compared to “PR”, “AR” alignment increased rV95% for an average of 34.4% across all patients. rV95% (cc) averaged over all fractions was significant from planning values for 10/13 patients for “PR” and for 9/13 for “AR”. 3/13 patients had reproducible anatomy. Of patients with non‐reproducible anatomy, three had dosimetrically more favorable, while seven had less favorable anatomies. Most shift differences (82.3%) between the “PR” and “AR” alignments larger than 2 mm resulted in rV95% changes larger than 2 cc. Most shift differences (82.2%) of 2 mm or less between the “PR” and “AR” alignments resulted in rV95% changes less than 2 cc. The average percentage of fractions among patients in which anterior or posterior shifts for “AR” and “PR” alignment was larger than the PTV margins was 9.1% (0.0%–37.5%) and 1.3% (0%–10%).

**Conclusion:**

Rectal deformation and subsequent inconsistent interfraction separation between prostate and rectal wall translate into anatomical changes that cannot always be mitigated with rigid alignment. If systematic differences exist due to a non‐reproducible planning anatomy, attempts to restore the planned rectal doses through anterior rectum alignment produce rather small improvements and may result in unacceptable target underdosage.

## 1 INTRODUCTION

As dose escalation in localized prostate cancer has become paramount for reduction in biochemical and clinical failure,^1^, ^2^, ^3^, ^4^, ^5^, ^6^ so too has the use of smaller and more conformal radiotherapy treatment fields. This necessitates a clear understanding of the localization techniques for the relevant regional anatomy in order to minimize and mitigate the target and organs at risk positional uncertainties. It has been reported that highly conformal treatments in conjunction with on‐line corrections are useful given the biochemical progression‐free survival benefit and improvement in rectal toxicity.^7^, ^8^, ^9^ Various methods for on‐board alignment have been explored and their performance has been quantified by numerous groups. Most commonly, the prostate is aligned either by matching intraprostatic fiducial markers or prostatic soft tissue (with or without aid from the planning contours) on daily cone beam computed tomography (CBCT). Each has advantages and disadvantages. Marker placement is an invasive procedure, and, in theory, the fiducials are susceptible to migration, though the observed migrations are regarded as negligible.^10^, ^11^, ^12^, ^13^, ^14^, ^15^, ^16^ On the upside, fiducials are easily visualized with either planar or volumetric imaging, and their alignment is consistent and reliable,^17^ thus making the alignment more reproducible. The soft tissue alignment has larger variability^18^, ^19^ due to relatively poor image quality (contrast‐to‐noise ratio of CBCT is lower, reducing the visibility of the soft tissue) and deformations. In addition, CBCT imaging requires prolonged treatment time (that may be further augmented by the need to have the alignment validated by a physician prior to treatment), and additional (albeit likely clinically insignificant) radiation exposure.^20^ CBCT provides the benefit of being able to visualize the neighboring anatomy and detect soft tissue changes like prostate deformation, bladder filling and rectum distension. When using CBCT, the soft tissue alignment based on gray values underperforms compared to manual alignment to fiducial markers.^10^ Contour‐based alignments that guide the match by projecting the planning contours onto the CBCT have been successful, but inferior to the fiducial alignment.^18^, ^21^ The comparison between soft‐tissue without contour‐guide and fiducial‐based alignments returned rather consistent results that show the methods have uncertainties of the same order of magnitude, but do favor the marker‐based alignment.^10^, ^22^, ^23^, ^24^ Li et al.^25^ evaluated dual marker‐based and soft‐tissue‐based image guidance and found that marker‐based alone is not accurate enough and additional adjustments are needed for some patients using CBCT. Regardless, CBCT manual soft‐tissue alignment is deemed acceptable, even if potentially inferior to fiducial alignment.

IGRT aims primarily at ensuring that the treatment target receives the prescribed dose more accurately due to the ability to reduce residual positional uncertainties in the delivery of the treatment. The prostate, however, has two critical organs in its close proximity—the bladder and the rectum, with the anterior aspect of the rectum wall being the major dose‐limiting structure. As such, there has been a sustained interest and effort to evaluate how the daily rectal variability translates into dosimetric deviations from the planned values in the delivery of highly conformal radiation treatments; an endeavor made possible by the availability of volumetric anatomical image data from the use of IGRT. The patient population was heterogeneous across the studies, with some institutions enforcing strict bowel preparation protocols for simulation and treatment,^26^, ^27^, ^28^, ^29^, ^30^, ^31^, ^32^, ^33^ while others only required this for simulation^34^, ^35^ or neither for simulation nor for treatments.^36^, ^37^, ^38^, ^39^, ^40^, ^41^, ^42^ The Supplemental Table summarizes observations regarding rectal doses from several studies.

Most studies focused on reporting the cases where the rectal doses exceeded the planned values, but a closer look at the data reveals that fraction doses lower than the planned values may occur as well. While sparing the rectum may improve the quality of life, it is important to recognize the recent findings by Marcello et al.^43^ from analyses over three randomized phase 3 trials (RADAR trial,^44^, ^45^ MRC RT01 trial^46^ and CHHiP trial^47^) regarding the increased risk of disease progression if the coverage of the posterior prostate is not adequate.

To summarize the vast literature available to date—appropriate coverage of the prostate is achievable with IGRT, but simultaneously constraining rectal doses that are consistent with planned values is challenging. No interpretation emerged from the reviewed studies regarding the specific circumstances causing the changes in rectal doses, beyond an implied daily random variation, with most studies only focused on reporting the increase in rectal doses over the treatment duration.

It must be noted that in the case of prostate irradiation, the local anatomy of interest changes non‐rigidly. Not only do the structures of interest deform (for example due to rectal filling variability), but the relative position between these structures varies as well. This is important, because on‐board image guidance is executed by applying rigid translational and rotational corrections.

To this end, our goal was to investigate if the rigid on‐board alignment can fulfill the rectum planning goals by estimating the range for the delivered rectal doses between the doses corresponding to two alignment endpoints—always align the prostate (scenario “PR”) and always align the anterior rectum (scenario “AR”). The anterior rectum (AR) as used in this paper is the external portion of the rectum facing the prostate. The AR is not defined by anatomical landmarks, but as the extent of the rectum that lies along posterior prostate. We sought to identify the general patterns that describe the anatomical variability (random and systematic) and its consequences on the daily doses, and we evaluated the relationship between the magnitude of the alignment shifts from “PR” to “AR” and the change in the corresponding rectal dose (i.e., rV95%), in a first report of this kind, to our best knowledge.

## 2 METHODS AND MATERIALS

### 2.1 Patient population, CT scanning and contouring

Thirteen patients with implanted fiducials (intraprostatic or at the prostate bed site after resection) undergoing moderately hypofractionated or conventionally fractionated radiotherapy for localized prostate cancer were retrospectively reviewed. Treatments were delivered in the supine position and were created and optimized using Varian Eclipse (Varian Medical Systems, Palo Alto, CA). Prior to CT simulation and for all treatments, patients were instructed to maintain approximately similar bladder and rectal filling by liquid intake and bowel movement prior to treatment. Various degrees of rectal distensions were exhibited at the time of the planning CT scan acquisition and patients were not re‐scanned due to rectal filling variations. The planning CT consisted of 3 mm slices acquired in helical mode, extending from the top of the iliac crests superiorly to the perineum inferiorly. The CTV included the prostate or prostate bed, as well as the seminal vesicles and the pelvic nodes, when applicable. The planning target volume (PTV) was designed to be a 4−7 mm margin in all directions from the CTV with some posterior margins less, typically 4 or 5 mm. The rectum was contoured on CBCT images following the same RTOG pelvic normal tissue contouring guidelines^48^ as for the contouring for treatment planning, which spanned in length from the ischial tuberosity inferiorly to the rectosigmoid flexure superiorly.

### 2.2 Daily alignment scenarios

#### 2.2.1 Treatment alignments

Before each treatment, all patients had daily image guidance consisting of daily planar kV. CBCT was used either daily or weekly. During setup for treatment, bladder and rectal filling was assessed using CBCT. While attempts were made to match the rectal and the bladder filling to planning CT (rectum voiding, waiting period for bladder filling), patients were treated even in cases of deviation after attempts had been made to correct bladder and bowel filling. A patient‐dependent hybrid alignment was used, that consisted of the alignment to fiducials with consideration to rectal deformation. The daily evaluation of the dose to the target based on the alignment of the day was not the purpose of our study.

#### 2.2.2 Study alignments

A total of 239 CBCTs from this cohort of patients were evaluated for this study. The number of fractions analyzed per patient was between 6 and 37 (median 19). CBCTs were rigidly registered with the planning CT scans using MIM Software (MIM Software Inc., Cleveland, OH, USA) and shifts were recorded for two different alignment strategies: correction to align the prostate (“PR”), and correction to align the anterior rectum (“AR”).

The prostate alignment “PR” was executed by aligning the fiducial markers inside the prostate. The relative position of these markers may randomly change daily due to, for example, small prostate deformations; thus, the alignment of the markers was achieved by splitting the differences among the three markers, as assessed by visual inspection.

**FIGURE 1:**
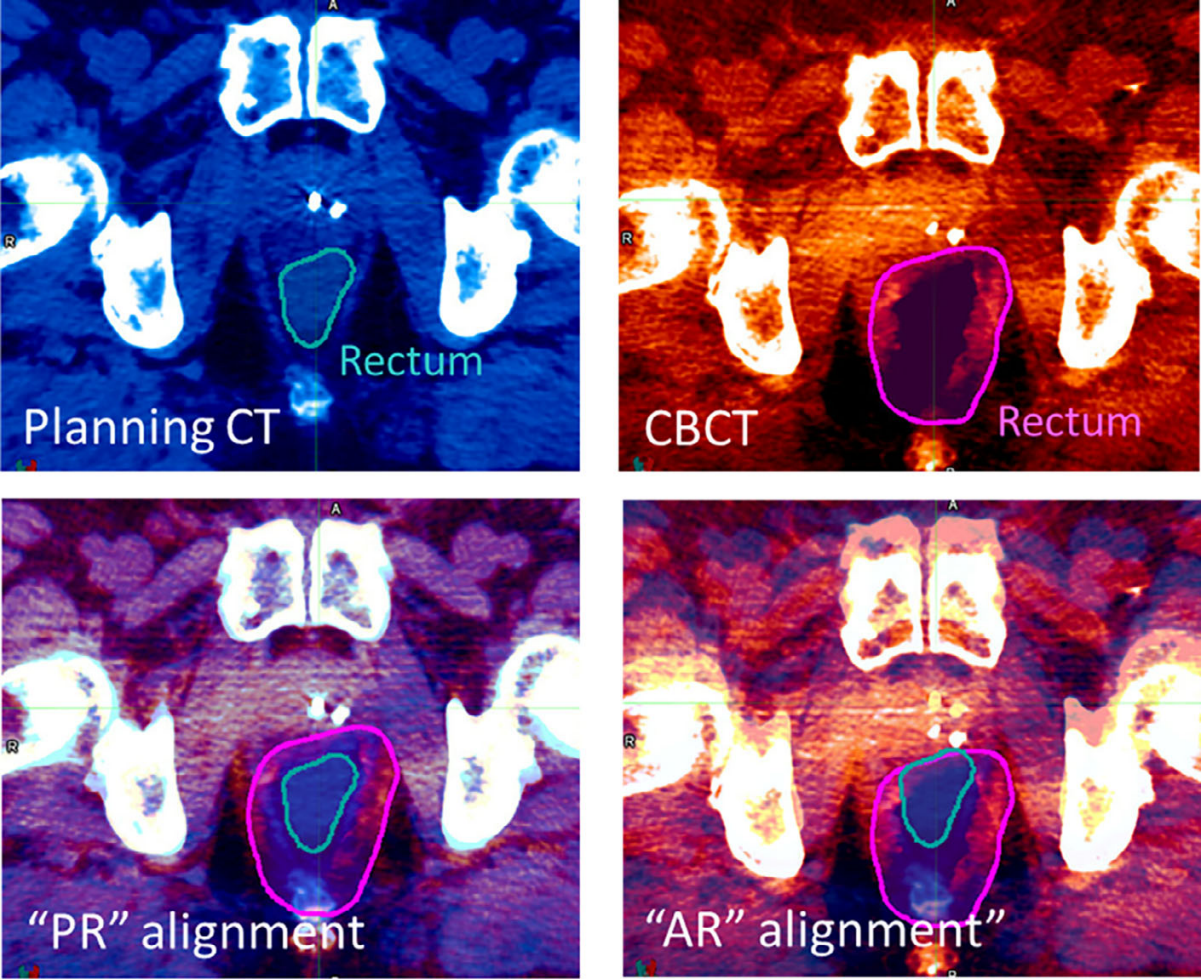
Prostate (“PR”) versus anterior rectum (“AR”) alignment. The rectum is contoured on the planning CT and on the daily CBCT (top row). The “PR” alignment is executed by aligning the fiducial markers inside the prostate. The “AR” alignment aims to align the anterior wall of the rectum. Note no perfect alignment is possible for either “PR” or “AR”, due to the non‐rigid nature of the involved anatomy. CBCT, cone beam computed tomography.

The anterior rectum alignment “AR” aligned the anterior rectum as seen on CBCT and the anterior rectum from the treatment planning CT. Nonetheless, due to the non‐rigid nature of the changes, a perfect alignment is never possible; instead, the goal was to carry out the best alignment as assessed by visual inspection of the anterior rectum in the vicinity of the prostate, much like this would be performed at the treatment console. Example “PR” and “AR” alignments are shown in Figure 1.

### 2.3 Dose mapping and rectal dose variation

The rectal doses received in each alignment strategy were estimated by rigidly mapping the planning dose on each daily CBCT according to the respective shift of each alignment strategy.^30^, ^37^, ^49^ The metric evaluated in our investigation was the rectal volume receiving 95% of the prescribed dose, as volumes enclosed by higher isodose lines (IDLs) are more susceptible to alterations due to geometrical changes along the beam axis in highly conformal plans. Note that the trends observed for rV95% are expected to be equally applicable for any other dose levels.

The choice of a more common dose‐volume parameter (e.g., V25–V40, V50–V60, V70–80, that Stenmark et al,^50^ for example, suggested to be associated with bleeding, incontinence, and overall bowel problems) was not a practical choice for our study because the patient population was heterogeneous in terms of prescription dose and fractionation.

The daily and average (over all fractions with CBCT alignments) differences between rV95% for the “PR” and “AR” alignments were evaluated as an indicator of which alignment was more effective at sparing the rectum for each patient. The differences between “PR” and “AR” alignments in essence characterize the *random* aspect of the daily changes in local anatomy.

**FIGURE 2:**
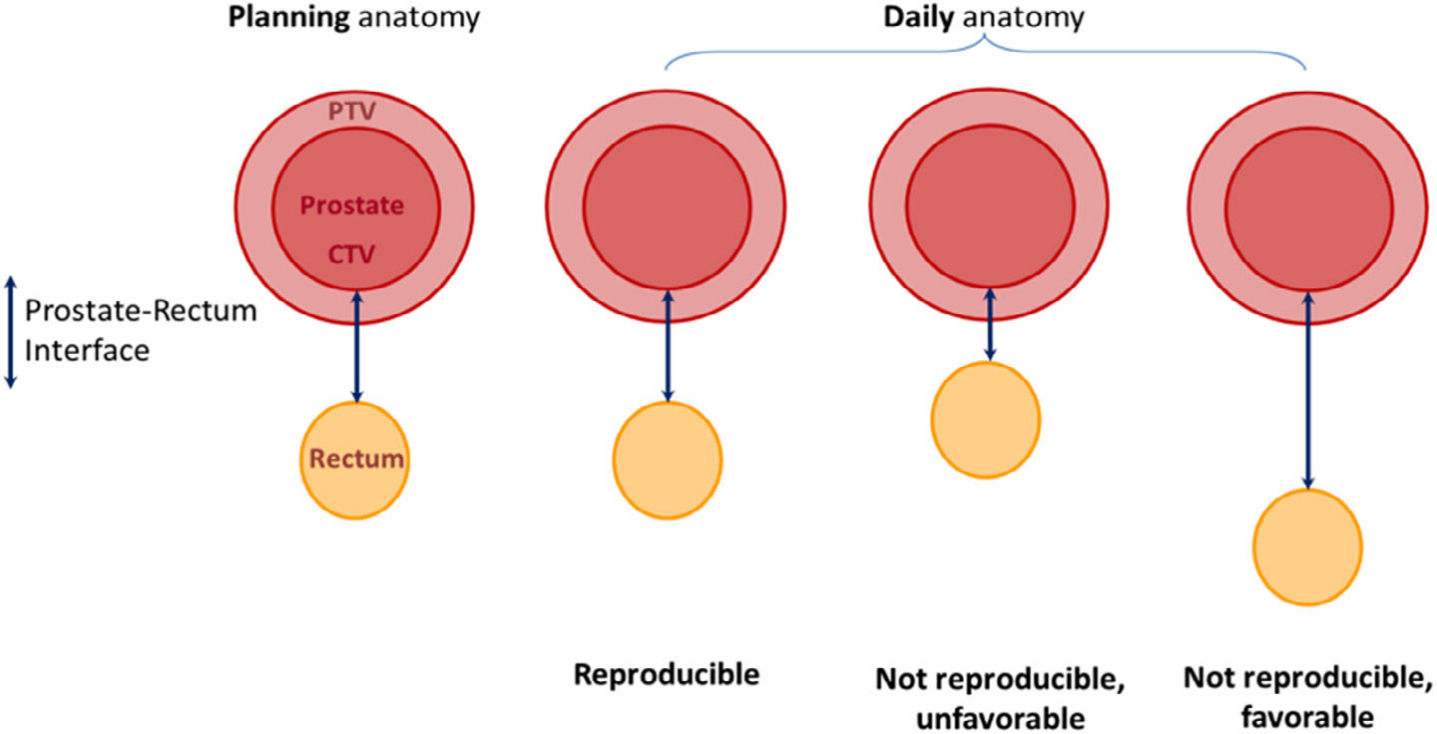
Classification of the topological favorability for rectum depending on the daily separation between the prostate and the rectum relative to the planning configuration: reproducible, not reproducible and unfavorable, not reproducible but favorable.

We also assessed the difference between the “PR” rV95% and the planning rV95%. This difference can reveal *systematic* daily differences in the local anatomy of interest. Figure 2 illustrates the three scenarios that can occur daily:

- Increases in the “PR” rV95% indicate a not reproducible and unfavorable daily anatomy, where the separation between the prostate and the rectal wall decreases and the dose to the rectum increases;
- Decreases in the “PR” rV95% indicate a not reproducible but favorable anatomy, where the separation between the prostate and the rectal wall increases and the dose to the rectum decreases;
- The planning anatomy was considered reproducible if the daily rV95% was within ±2 cc from the planning anatomy. This value does not originate in any established clinical basis, but the maximum dose to 2 cc is usually considered to be the appreciable maximum dose in organs at risk. Note also, for example, that if two individuals contour a rectum assumed to be a cylinder of 15 cm length and 3.4 cm radius, then the average difference between axial contours between the two users would have to be within 0.1 cm for the difference between the two rectum volumes to be 2 cc; that is, 2 cc is a good measure for a sub‐millimeter inter‐user contouring reproducibility and indicates that a 2 cc threshold for reproducibility is a pertinent choice.

The comparison between “AR” and planning rV95% was also conducted.

The deviations in the daily anatomy from the planning anatomy were also described by the percentage of fractions during which treatment alignment resulted in higher rectal doses and by the distribution of prostate‐rectum separation changes in 5 mm increments, over all patients and all fractions. The separation changes were determined by measuring the distance between each fiducial and the most anterior portion of the rectum using the ruler function in MIM for the initial planning CT and each subsequent CBCT. The value was computed as the difference between the average distance of each fiducial to the anterior rectum between the planning and each CBCT.

**FIGURE 3:**
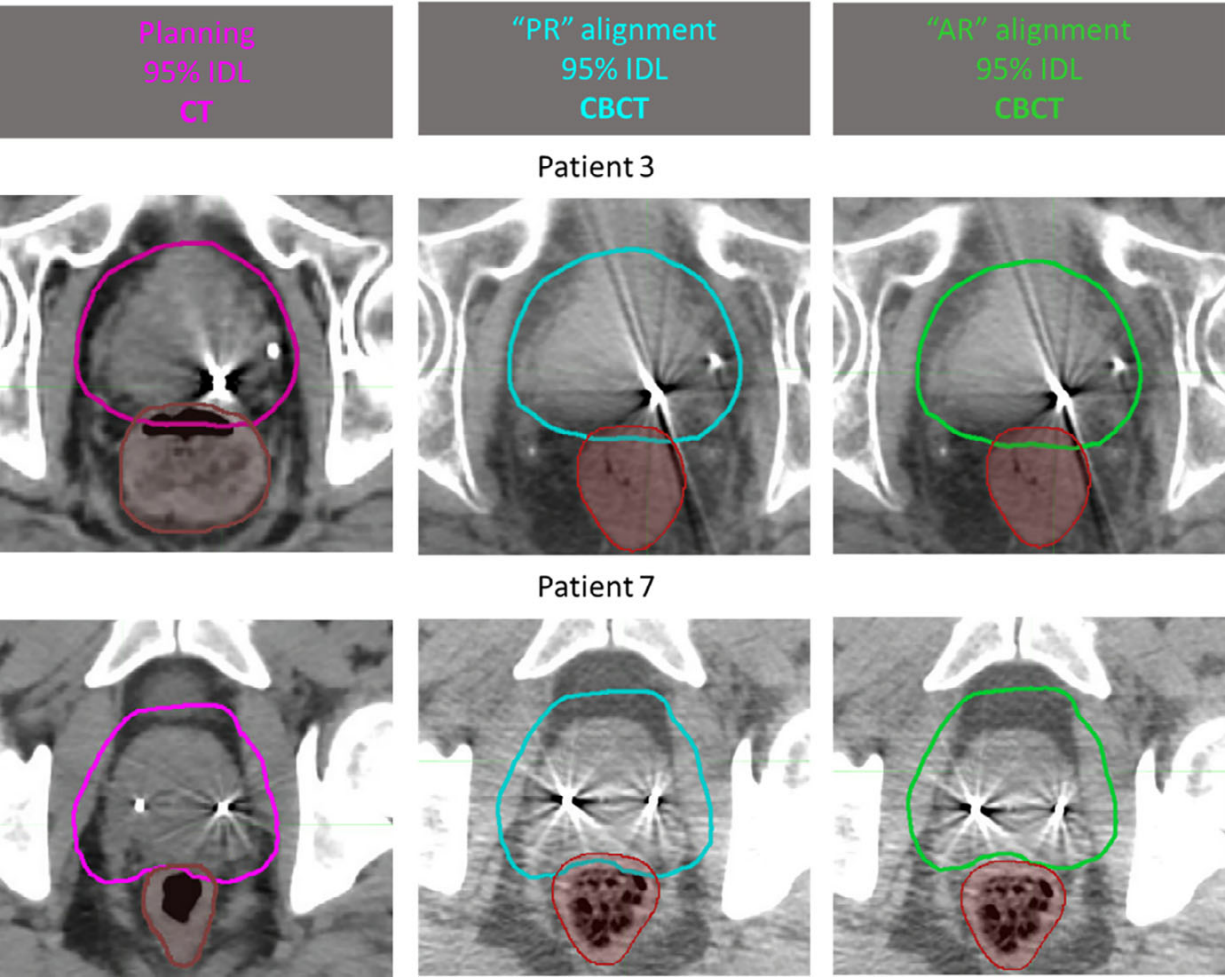
Example “PR” versus “AR” alignments with 95% IDL projected on the planning CT and daily CBCT. For the “PR” alignment (teal), the marker seeds are always at about the same location relative to the 95% IDL as the planning anatomy (pink). Brown—Rectum contour on the planning CT. Red—Rectum contour on the CBCT. **Patient 3**: IDL95% = 62.7 Gy. The rectum of the day as seen on CBCT is less distended compared to planning, thus further away from the prostate—the anatomy of the day is *favorable*, compared to planning. “PR” alignment: rectum receives less dose compared to planning. “AR” alignment: the anatomy has to be shifted anteriorly from the “PR” alignment and the *anterior* border of the prostate is *underdosed* compared to planning. Rectum receives more dose if “AR” alignment is executed, compared to “PR”. **Patient 7**: IDL95% = 57 Gy. The rectum of the day as seen on CBCT is more distended compared to planning, thus closer to the prostate—the anatomy of the day is *unfavorable*, compared to planning.“PR” alignment: rectum receives more dose compared to planning. “AR” alignment: the anatomy has to be shifted posteriorly from the “PR” alignment and the *posterior* border of the prostate is *underdosed* compared to planning. Rectum receives less dose if “AR” alignment is executed, compared to “PR”. IDL, isodose lines.

In addition to that, the percentage of fractions where the magnitude of shifts of the rectum in the anterior or posterior direction for “AR” alignment from the “PR” aligned anatomy were larger than the anterior and posterior PTV margin respectively were recorded. This is important because, assuming that the isocenter is located inside the prostate, rectum shifts away from the isocenter (i.e., translation posteriorly) by an amount that exceeds the posterior PTV margin could underdose the posterior aspect of the prostate, whereas shifts towards the isocenter (i.e., translation anteriorly) by an amount larger than the anterior PTV margin could underdose the anterior aspect of the prostate (Figure 3).

Last, we investigated the magnitude of rV95% changes as a function of the shift between “PR” versus “AR” alignments.

### 2.4 Statistical analysis for rV95% and “PR” versus “AR” shifts

The anterior‐posterior shifts for both “AR” and “PR” alignments were recorded for each CBCT. In addition, rV95% was recorded for the “AR” and “PR” alignment as stated above. To determine the statistical significance of the observed changes a one‐tailed *T*‐test was used with *p* = 0.05 significance level. It was assumed that the two different alignment strategies were independent and approximately normally distributed with similar level of variance. The null hypothesis was that there is no difference between the mean of the rV95% for “AR” versus “PR” alignments.

## 3 RESULTS

### 3.1 Daily rectum dose variability

Figure 3 illustrates two examples of the 95% IDL for the planned dose (on CT) and the daily dose (on CBCT) for the alignment strategies investigated. These examples outline two distinct scenarios.

#### 3.1.1 Non‐reproducible, but favorable daily anatomy

For Patient 3, the rectum of the day is further away from the prostate, thus the daily anatomy is *favorable* compared to the planning anatomy. In the “PR” alignment the rectal wall would receive less than the planned dose. For the “AR” alignment, the anatomy would need to be shifted anteriorly from the “PR” alignment and the anterior border of the prostate would be underdosed compared to planning. The rectum receives more dose if the “AR” alignment is executed, compared to “PR”.

#### 3.1.2 Non‐reproducible and unfavorable daily anatomy

For Patient 7, the rectum of the day is closer to the prostate, thus the daily anatomy is *unfavorable* compared to the planning anatomy. In the “PR” alignment the rectal wall would receive more than the planned dose. Consequently, for the “AR” alignment the anatomy would have to be shifted posteriorly from the “PR” aligned position and the posterior border of the prostate would be underdosed compared to planning. The rectum receives less dose if the “AR” alignment is executed, compared to “PR”.

**FIGURE 4:**
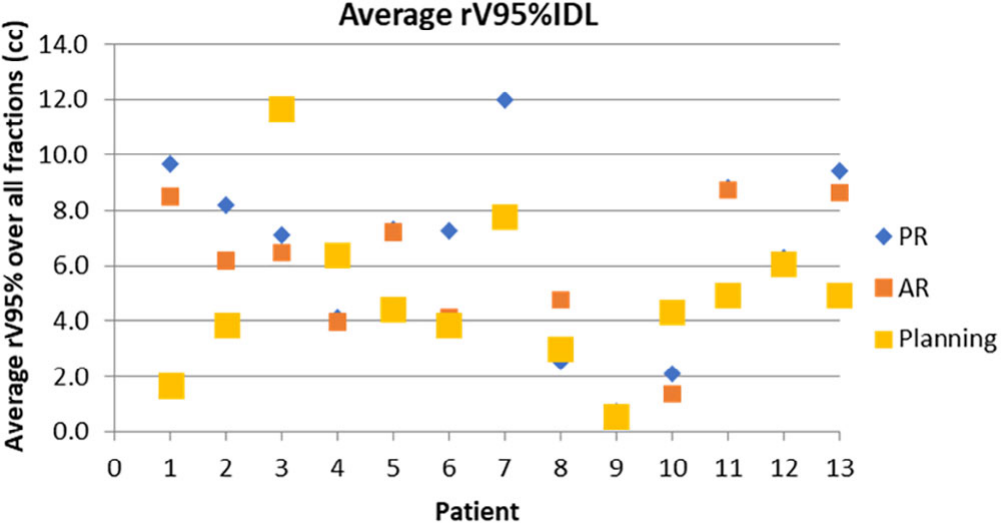
The average rectal V95% for planning CT, “PR” and “AR” alignment for each patient.

Figure 4 shows the planning rV95% (cc) and the average rV95% (cc) over all available fractions for each patient, for each alignment strategy. Table 1 includes the corresponding ranges and the significance level of the differences between these “PR”, “AR” and planning values for each patient.

**TABLE 1:** rV95% for each patient: Average over all fractions and significance of the differences, ranges.

Patient ID	rV95% at planning (cc)	Average rV95% over all fractions (cc)	Range (cc)	Average rV95% over all fractions (cc)	Range (cc)	“PR” versus “AR”		Planning versus “PR”		Planning versus “AR”	
		“PR”	“PR”	“AR”	“AR”	*p*‐value	significance	*p*‐value	significance	*p*‐value	significance
1	**1.7**	**9.7**	1.2–30.8	**8.5**	1.3–30.2	0.31959	N	0.00005	Y	0.00005	Y
2	**3.9**	**8.2**	1.2–9.6	**6.2**	1.4–15.7	0.10590	N	0.00030	Y	0.01995	Y
3	**11.7**	**7.1**	2.8–15.7	**6.5**	3.9–15.2	0.27180	N	<0.00001	Y	<0.00001	Y
4	**6.4**	**4.1**	2.0–6.7	**4**	0.6–7.7	0.43990	N	0.00189	Y	0.00002	Y
5	**4.4**	**7.3**	1.3–14.5	**7.2**	1.1–13.8	0.46120	N	0.00177	Y	0.00407	Y
6	**3.9**	**7.3**	3–5.1	**4.1**	4.6–8.1	0	Y	<0.00001	Y	0.27006	N
7	**7.8**	**12**	1.9–24.8	**7.8**	4.0–33.8	0.17057	N	0.09844	N	0.43704	N
8	**3**	**2.5**	0.0–12.8	**4.7**	0.7–5.1	0.00305	Y	0.04183	Y	0.00984	Y
9	**0.6**	**0.7**	0.0–1.5	**0.4**	0.0–1.8	0.15890	N	0.34786	N	0.13141	N
10	**4.4**	**2.1**	0.2–3.0	**1.4**	0.1–3.0	0.25000	N	0.01275	Y	0.00001	Y
11	**5**	**8.9**	3.8–33.7	**8.7**	3.3–21.6	0.46830	N	0.00036	Y	0.00782	Y
12	**6.1**	**6.3**	0.6–15.0	**6.1**	1.7–15.4	0.41880	N	0.28823	N	0.41202	N
13	**4.9**	**9.4**	1.4–20.6	**8.7**	1.4–19.1	0.21719	N	<0.00001	Y	<0.00001	Y

### 3.2 Random and systematic variability in rV95% (cc) between planning, ”AR” alignment and “PR” alignment

#### 3.2.1 Random variability: “PR” versus “AR” rV95% (cc)

Compared to the “PR” alignment, the alignment to “AR” increased the rV95% for an average of 34.4% of the 239 fractions across all patients (range for individual patients: 0%–72% of their respective total number of fractions). The “PR” and “AR” alignments resulted in a significant difference between the average rV95% over all fractions for 2 out of 13 patients (Patient 6 and 8) at *p* < 0.05. The average rV95% over all fractions was larger with the “AR” alignment only for Patient 8.

#### 3.2.2 Systematic daily variability

*Planning versus “PR” V95%(cc)*: The rV95% (cc) averaged over all fractions and the corresponding planning value were significantly different for 10 out of 13 patients (Patient 1, 2, 3, 4, 5, 6, 8, 10, 11, 13) at *p* < 0.05.

*Planning versus “AR” V95%(cc)*: The rV95% (cc) averaged over all fractions and the corresponding planning value were significantly different for 9 out of 13 patients (Patient 1, 2, 3, 4, 5, 8, 10, 11, 13) at *p* < 0.05.

**FIGURE 5:**
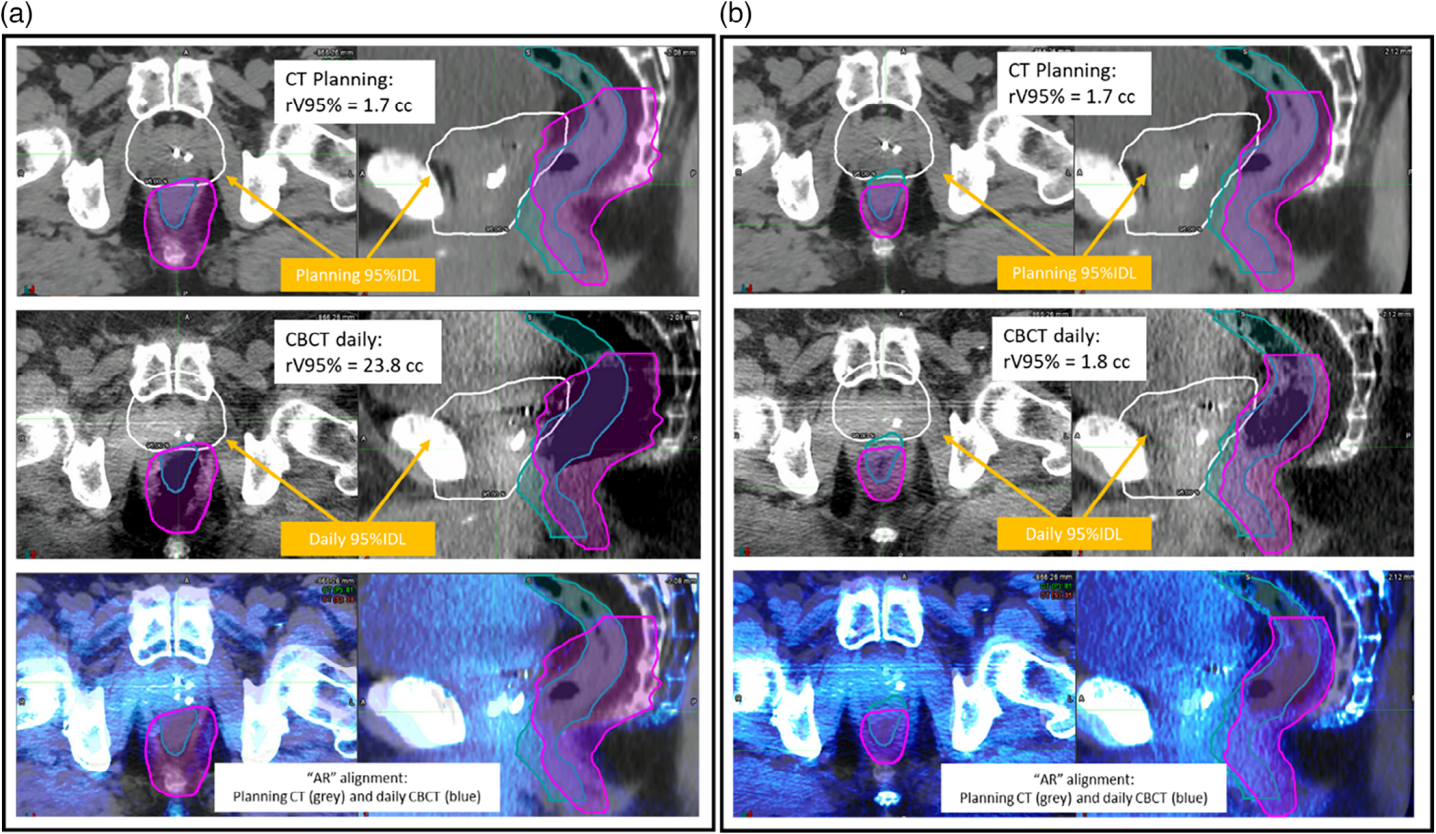
Daily (pink) and planning (teal) rectum contours and the corresponding rV95% IDLs (white). **(a)**: Example of daily scenario where “AR” rV95% exceeds the planning value (23.8 cc vs. 1.7 cc). **(b)**: Example of daily scenario where “AR” rV95% is similar to the planning value (1.8 cc vs. 1.7 cc). **Top row**: Planning CT and corresponding V95% IDL. **Middle row**: Daily CBCT and the corresponding V95% IDL. **Bottom row**: “AR” fusing of the planning CT and the daily CBCT. CBCT, cone beam computed tomography.

Of note for the “AR” alignment is that it does not always restore the rectum planning dose values, due to the shape and the distribution of the rectal filling which preclude a true rectal alignment on every slices. Figure 5 illustrates the “AR” alignment relative to the planning anatomy for two fractions, one where the “AR” was inferior dosimetrically to planning, and one where the planning dose was restored via “AR” alignment.

### 3.3 Planning anatomy reproducibility (“PR” rV95% vs. planning rV95%)

**FIGURE 6:**
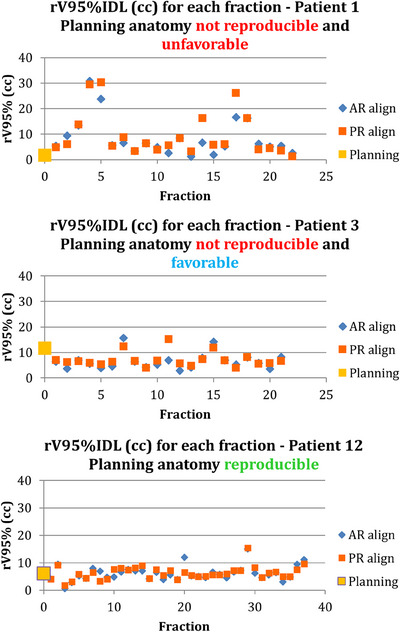
All fractions data for three patients that exhibited different overall anatomical changes over the course of their treatment. Patient 1: planning anatomy not reproducible and unfavorable; Patient 3: planning anatomy not reproducible and favorable; Patient 12: planning anatomy reproducible.

**FIGURE 7:**
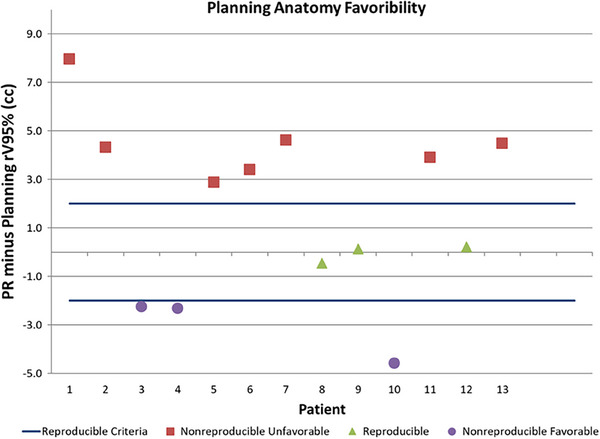
Classification of anatomical favorability based on the difference between the average “PR” rV95% and planning rV95%. Patients within ± 2 cc are considered reproducible. Patients with differences greater than 2 cc are nonreproducible unfavorable and patients with differences less than −2 cc are nonreproducible favorable.

Figure 6 shows rV95% data for all alignment scenarios and for all fractions for three example patients that exhibited different overall patterns for anatomical changes over the course for their treatment: Patient 1 ‐ planning anatomy not reproducible and unfavorable; Patient 3 ‐ planning anatomy not reproducible and favorable; Patient 12 ‐ planning anatomy reproducible. Of all 13 patients from this study, 3 had a reproducible anatomy (Patients 8, 9 and 12), 7 had not reproducible and unfavorable anatomy (Patients 1, 2, 5, 6, 7, 11, 13), and 3 had not reproducible but favorable anatomy (Patients 3, 4, 10). In percentages, the planning anatomy was considered reproducible in 23.1% of patients, with an additional 23.1% being not reproducible, but with favorable anatomy. The remaining half of patients were classified as unfavorable. The “PR” minus planning rV95% classified as described above is shown in Figure 7.

### 3.4 Distribution of the prostate—Anterior rectum separation

**FIGURE 8:**
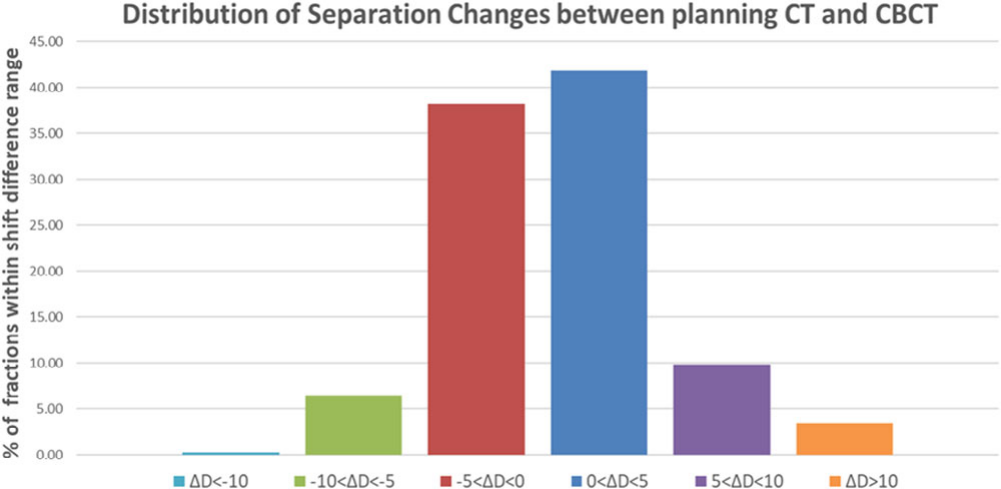
The average anterior rectum separation difference between daily (CBCT) and planning (CT) values in AP direction, categorized in 5 mm increments for all patients. ΔD < 0 indicate that the daily separation is less than the planning separation, and the rectum is closer to the prostate. ΔD > 0 indicate that the daily separation is more than the planning separation, and the rectum is further away from the prostate.

The distribution of the separation changes between the posterior prostate and the anterior rectum was tabulated in 5 mm increments and it is illustrated in Figure 8, using the daily data from all patients and all fractions. 77.2% of fractions were within ± 5 mm of the average distance of the fiducials, with 3.6% of fractions having a distance larger than ± 10 mm.

### 3.5 Evaluation of rV95% changes between “PR” and “AR” relative to the difference in shift between the two alignment strategies

**FIGURE 9:**
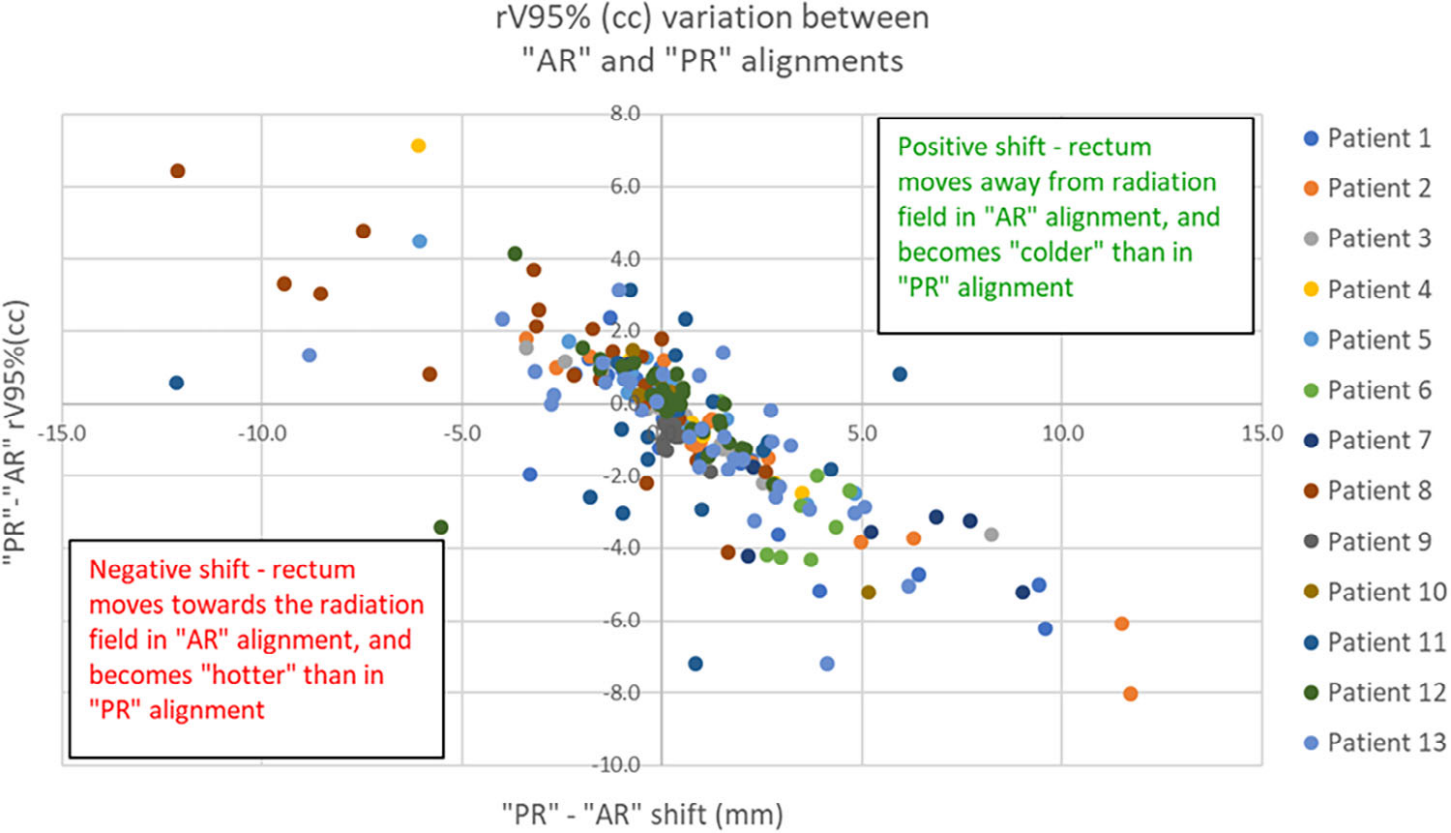
Distribution of the changes in rV95% (cc) from PR to AR alignments relative to the shift from the PR to the AR alignment.

The distribution of the changes in rV95% from “PR” to “AR” alignments relative to the shift from the “PR” to the “AR” alignment is illustrated in Figure 9. Considering our 2 cc level of significance in differences of rV95%, we found that most shift differences (82.3%) between the two scenarios larger than 2 mm resulted in rV95% changes larger than 2 cc. Most shift differences (82.2%) of 2 mm or less resulted in rV95% changes of 2 cc or less.

### 3.6 Analysis of shift magnitude versus PTV margin

Starting from the “PR” aligned anatomy, the average percentage of fractions in which the posterior shifts for “AR” alignment were larger than the PTV margins was 9.1% (0.0%–37.5%).

Starting from the “PR” aligned anatomy, the average percentage of fractions in which the anterior shifts for “AR” alignment were larger than the anterior PTV margin was 1.3% (0%–10%).

**FIGURE 10:**
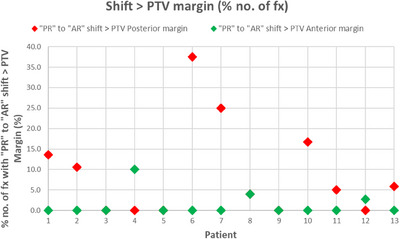
Percentage of treatments where the shifts used for treatment were greater than the PTV margin. Due to anisotropic PTV margins, the shifts are characterized as exceeding the margin during a fraction as defined in the anterior or posterior direction. The percentages are shown for each patient.

The percentage of shifts greater than the PTV margin for each patient are shown in Figure 10. No patients had “AR” shifts larger than PTV margin more than 50% of the time and 4/13 patients had fractions which increased the rV95% more than 50% of the time.

## 4 DISCUSSION

The daily data analysis for the population considered in this study suggests that most often the two alignment strategies do not result in significantly different rV95% when averaged over all delivered fractions, despite the non‐rigid local anatomical changes. The more important differences are expected to exist between the planning rV95% and either the “PR” or the “AR” corresponding values, underscoring the observation that systematic deviations are expected to be more consequential than the random variations.

The “PR” and “AR” alignments were evaluated visually, and, in all cases, the achieved alignments were deemed appropriate. While the quantitative results reported in the subsequent sections would change as a result of inter‐ and/or intra‐user variability (since both alignments investigated are ultimately validated subjectively), it is unlikely that the general findings from our study will change.

The reproducibility aspect of the local anatomy of interest becomes especially important if the rectum sparing takes precedence over prostate coverage in any particular clinical scenario. As shown above, if the daily anatomy is such that the rectum is farther away from prostate compared to planning (e.g., Patient 3, Figure 5), daily rectal sparing is not a concern, because this scenario implicitly spares the rectum even more than what was designed during treatment planning.

However, if rectal wall sparing is to be relevant, several aspects must be considered. First, ideally, the sparing must be deemed meaningful from a clinical standpoint. Our analysis seems to suggest that shifts of about 2 mm or less most likely will result in volume changes less than 2 cc for rV95%. Second, in principle, the “AR” shift should not exceed the PTV margin, to avoid target underdosage. While the prostate coverage was not assessed in this paper, PTV margins have been historically defined using random and systematic variations and exceeding these variations could affect target coverage. This hypothetical target underdosage would be potentially more consequential if the target coverage was not 100% at the time of planning, more so perhaps if the treatment is hypofractionated, since at higher fraction dose the underdosage represents a larger percentage of the prescribed dose, and, with fewer fractions, the chance of recovering random underdose by random overdose is less.

**FIGURE 11:**
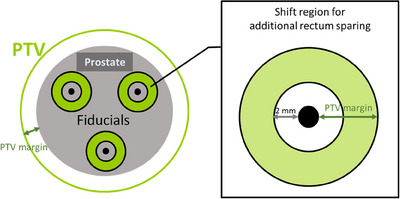
Practical implementation of the additional rectal sparing achievable daily.

As a practical implementation of an IGRT workflow that maintains the prostate coverage but maximizes the rectal sparing, one could consider a two‐step process that utilizes a doughnut‐shaped contour around the makers seeds, with the inner radius 2 mm (based on the above observations) and the doughnut thickness equal to the size of the PTV margin, as shown in Figure 11. The daily alignment would start by aligning the prostate target using the fiducial markers, and the additional rectal sparing would be performed such that the fiducials stay confined inside the doughnut boundaries (in principle, lowering the couch down only when patients are in supine position). We reiterate that the additional rectal sparring for a given daily anatomy is possible only in the case of an unreproducible and unfavorable anatomy, as explained earlier; the cases with “reproducible” or “unreproducible but favorable” anatomy cannot benefit from additional rectal sparing attempts by aligning the daily anterior rectal wall to the anterior rectal wall from the planning CT.

The current study demonstrates that interfraction motion and deformation of the rectum/prostate with on‐line correction of rigid prostate translations can potentially have unintended dosimetric consequences. Over all patients and all fractions, on average ∼10% of alignment shifts (anterior and posterior cumulated) from “PR” alignment to incorporate the alignment of prostate‐rectal interface were larger than the PTV margin, potentially resulting in underdosage of the prostate. Shifts anteriorly that exceeded our anterior PTV margin were not common, with the additional 1−2 mm anterior margin capturing most daily anatomy changes.

The goal of this study is not to recommend a specific PTV expansion, but we do note that both anterior and posterior margins appear to account for daily anatomical changes between both alignment strategies.

The “AR” alignment resulted in an increase in the daily rV95% compared to “PR” alignment in ∼35% of the 239 fractions reviewed. However, when averaged over all fractions for each patient, rV95% was larger for the “AR” alignment only for one patient.

The average rV95% over all patients and fractions was slightly less for the “AR” alignment compared to the “PR” alignment (5.7 cc vs. 6.6 cc, or about 13%, on average). The differences between “AR” and “PR” alignment were much less compared to the dosimetric differences between simulation planning anatomy and the “PR” aligned anatomy, which increased rV95% on average by about 35% (4.9 cc vs. 6.6 cc). This points to the observation that neither alignment can overcome non‐rigid daily variations and that the rectal deformation will greatly affect the dosimetry as compared to the original plan.

Our metric, the rV95%, is not common, but appears to be consistent with previously published literature in both the increase in rectal dose and the magnitude of the increase. Huang et al.^30^ found that the rectal volume increased by 36% on average compared to the planning CT, which translated to an increased mean dose of 22% on average. Hüttenrauch et al.^39^ saw a large range of increase in the D_V50%_ with some patients receiving a dose 50% higher than planned, however they did not describe any bowel preparation in their study and this could be a cause of the higher dose to the rectum compared to the planning CT. As outlined in the introduction, the body of literature surrounding prostate radiotherapy reveals a large amount of heterogeneity among studies. There are differences in fractionation, imaging, alignment, bladder and bowel preparation, and many other factors that can greatly affect comparison between studies. However, it is well known that the rectum can experience significant volume changes during the course of radiotherapy for prostate cancer, with some studies estimating varying rectal volumes ± 30% compared to planning CT.^51^

It was also observed that most patients exhibit mostly random variation in rectal volumes with rectums more distended on average during treatment compared at the time of simulation.^34^ Other studies have shown that in the majority of fractions (∼70%), prostate and rectal deformation were small enough that patient repositioning using soft tissue alignment strategies were reasonable to correct for interfraction variations.^26^ The study's authors note that better on‐line correction techniques may be needed when non‐rigid anatomical changes are present. This could include on‐line re‐planning but could also include alignment techniques that seek to minimize dosimetric differences when anatomical deviations, exist as we suggested earlier.

With low rates of rectal toxicity in the modern era with IMRT treatments and daily online soft‐tissue based IGRT using fiducials and CBCT, the clinical relevance of the dosimetric differences observed in the current study is called into question when treating with conventionally fractionated or moderately hypofractionated treatments. Emerging data about extreme hypofractionation studies supports its efficacy with low rates of rectal toxicity. However, at present the majority of patients treated on phase I/II trials had real‐time intrafraction motion tracking and small posterior margins.^52^ As this modality becomes more commonly used for patient convenience factors, the landscape of prostate IGRT will continue to adapt and evolve, and a clear understanding of the limitations of IGRT in the face of non‐rigid daily anatomical variations is important.

## 5 CONCLUSION

Rectal deformation and subsequent inconsistent interfraction separation between the prostate and the rectal wall translate into anatomical changes that cannot always be mitigated with rigid alignment. If systematic differences exist due to a non‐reproducible planning anatomy, attempts to restore the planned rectal doses through anterior rectum alignment produce rather small improvements and may result in unacceptable target underdosage.

## AUTHOR CONTRIBUTIONS

Mitchell Polizzi contributed to data collection, analysis and writing of the manuscript. Elizabeth Weiss contributed to study design, supervision of the project, and writing of the manuscript. Nuzhat Jan contributed to dataset contouring and review of the manuscript. Anthony Ricco contributed to dataset contouring and review of the manuscript. Alfredo Urdaneta contributed to study design, supervision of the project, and review of the manuscript. Mihaela Rosu‐Bubulac contributed to data collection, study design, analysis, supervision of the project, and writing the manuscript. All authors discussed the results and contributed to the final manuscript.

## CONFLICT OF INTEREST STATEMENT

The authors declare no conflicts of interest.

## Supporting information

Supporting Information

## Data Availability

Research data are stored in an institutional repository and will be shared upon request to the corresponding author.
