# Supplementary material for: Rectal deformation management with IGRT in prostate radiotherapy: Can it be managed with rigid alignment alone?
Source: J Appl Clin Med Phys. 2024 Jan 9;25(4):e14241. doi: 10.1002/acm2.14241 (PMC11005986; doi:10.1002/acm2.14241)
Supplement: Supplementary file 1 — Supporting Information [file ACM2-25-e14241-s001.docx]

Supplement

Summary of studies evaluating daily changes in rectal doses in prostate irradiation.

| Reference | Bowel preparation protocol at simulation and during treatment, and treatment modality | Observations regarding the delivered rectal doses | Alignment method |
| --- | --- | --- | --- |
| Peng C et al. 2011^26^ | Simulation – Yes  Treatment – Yes,  IMRT | Following prostate alignment, approximately 15% of the rectal V70Gy and V45Gy daily doses for the 20-patient population considered were larger than planned. Lower than planned doses were also recorded. | Prostate gland aligned using CT-on-rails. |
| Falco MD et al. 2011^27^ | Simulation – Yes  Treatment – Yes,  6-Field 3D-conformal | Rectal DVHs were lower than planned for all 6 patients reviewed. | Target aligned using CBCT. |
| Akin M et al. 2014^28^ | Simulation – Yes  Treatment – Yes,  IMRT and IMAT | None of the dose-volume histograms showed rectal V65≥17%; however, the rectal V40≤35% dose constraint (institutional acceptance criteria) was not met in 11 of the 20 patients investigated. | Surgical clip and soft tissue (prostate bed) as seen on CBCT. |
| McParland N et al. 2014^29^ | Simulation – Yes  Treatment – Yes,  IMRT | Significant differences in rectal doses for 4 out of 5 patients.  Except for one patient, the delivered dose was less than the planned dose. | Prostate tissue aligned using CBCT. |
| Huang T-C et al. 2015^30^ | Simulation – Yes  Treatment – Yes,  IMRT and VMAT | Large variations (increases or decreases) in the rectal volume for 28 patients (mean: 36%, range: 1-217%) and rectal doses (mean: 22%, range: 0-54%) despite bowel preparation regimens. | Isocenter aligned on CBCT for dose of the day recalculation. |
| Bostel T et al. 2019^31^ | Simulation – Yes  Treatment – Yes, IMRT | All DVH indices for target and OARs did not show significant deviations from the treatment plan for the 10 patients considered, when averaged over the entire population. However, considerable daily differences were noted for individual patients. | Prostate tissue aligned using daily in-room CT. |
| Nigay et al. 2019^32^ | Simulation – Yes  Treatment – Yes,  IMRT or VMAT | Cumulative daily rectal D15% was higher than planned for all patients and by more than 3 Gy for 4 out of the 5 cases investigated. | Intraprostatic fiducial alignments or local bony anatomy if prostate fossa or regional nodes were treated, using CBCT. |
| Hatton JA et al. 2011^33^ | Simulation – Yes  Treatment – Yes,  5-field 3D conformal | Rectal DVH results show a tendency to underestimate the delivered rectal dose, with 65% of all on-treatment plans for the 12 patients reviewed showing higher rectal doses than predicted. | Intraprostatic fiducials alignments using planar kV and CBCT twice weekly after treatment delivery. |
| Kupelian P et al. 2006^34^ | Simulation – Yes  Treatment – No,  Helical tomotherapy | Of the 10 patients, mean rectal V2Gy over the treatment course was less than planned for 1/10, as planned for 2/10 and larger for 7/10, with 2 cases exhibiting 5-10 cc difference. | Intraprostatic fiducial alignments using MVCT. |
| Chen L et al, 2010^35^ | Simulation – Yes  Treatment – No,  IMRT | Out of 20 patients, 28% of the treatment dose distributions did not meet the criterion of V40<35%, and 27% did not meet the criterion of V65 < 17%. | Weekly CT-on-rails scans used for dosimetric evaluations had BBs that were matched with the BBs from the planning CT. |
| Varadhan R et al. 2009^36^ | Simulation – No  Treatment – No,  IMRT | Rectal doses estimated weekly were, on average, larger than the planned ones for 4/5 patients, with differences as large as 12% observed. | Prostate tissue aligned via 3 gold seed markers using CBCT. |
| Sripadam R et al 2009^37^ | Simulation – No  Treatment – No,  4-field 3D conformal | For large/small planning rectal volumes, mean dose to the rectum was less/more than predicted. Rectal volume decreased over the treatment course in 78% of the daily CBCTs (for 13/15 patients). Typically, time of scan was p.m., treatment delivery in the a.m. | Prostate tissue aligned using CBCT. |
| Murthy V et al 2011^38^ | Simulation – No  Treatment – No,  Tomotherapy | Dose to 2 cc of the rectum and the absolute volume of the rectum and bladder receiving 100% and 70% of the prescribed dose exceeded the planning values in about 50% of the fractions analyzed. | Prostate aligned using MVCT. |
| Hüttenrauch P et al. 2014^39^ | Simulation – No  Treatment – No,  VMAT | Average rectum doses from half fractions used to evaluate delivered doses to rectum. Rectum was contoured on each CBCT such that the new contours were equal to the planning ones in terms of extension, since the cranial and caudal border of the contoured organ are not uniformly defined. The dose to the rectum was between 1% and 54% higher than predicted on the planning CT. | Match the anterior rectum wall and the bottom of the bladder between the CT reference data and the CBCT. |
| Pearson D et al 2016^40^ | Simulation – No  Treatment – No,  IMRT | Averaged over all 6 patients, V70 for rectum increased by approximately 4.5%, but looking at individual patients, 2/6 always had the daily dose exceed the planning dose, and 2/6 had always the daily dose less than the planned dose. | Prostate tissue aligned using CBCT. |
| Wahl M et al. 2017^41^ | Simulation – No  Treatment – No,  SBRT using Cyberknife | Some of the 10 patients analyzed demonstrated highly variable rectal position with substantially higher corresponding rectal dose, whereas others demonstrated relatively reproducible rectal position, as measured by V75%. | Intraprostatic fiducial alignments using MVCT. |
| Fuchs F et al 2019^42^ | Simulation – No  Treatment – No,  IMRT | Changes in rectal DVH were mostly affected by the rectal distension in the ventral direction. No trend was identified for daily doses for cases when endorectal rectal balloon (ERB) was used vs. cases when non-ERB cases, except that changes in the ERB group are slightly smaller. | Isocenter aligned on MVCT for dose of the day recalculation. |
